# Clinical determinants of social media use in patients with schizophrenia and schizo-affective disorder

**DOI:** 10.1192/j.eurpsy.2022.2027

**Published:** 2022-09-01

**Authors:** O. Elleuch, L. Zouari, N. Smaoui, I. Gassara, S. Omri, R. Feki, J. Ben Thabet, M. Maalej, N. Charfi, M. Maalej

**Affiliations:** 1 Hedi Chaker University Hospital, Psychiatry, Sfax, Tunisia; 2 Hedi Chaker University Hospital, Psychiatry C, Sfax, Tunisia

**Keywords:** Schizoaffective disorder, schizophrénia, clinical determinants, social media

## Abstract

**Introduction:**

Social media networks are becoming omnipresent in our lives, and more and more available to everyone including patients with mental illnesses.

**Objectives:**

Our study aimed to examine the prevalence of social media use in individuals with schizophrenia and schizoaffective disorder, and to examine the association of severity of symptoms with social media use.

**Methods:**

A total of 38 patients with schizophrenia or schizoaffective disorder were recruited from the outpatient unit of the department C of psychiatry in Hedi Chaker hospital of Sfax, Tunisia. Socio-demographic information as well as details about their social media use were collected from all the patients. Severity of schizophrenia symptoms was assessed on the Positive and Negative Syndrome Scale (PANSS). A logistic regression was used to explore the association between social media use and clinical characteristics of the participants.

**Results:**

Of the 38 study participants, 23.7% used social media. Facebook was the most popular social media site. The number of social media users were highest among participants aged 21–30 years old, married participants, residents of an urban region, employed participants and patients with a tertiary education level. Age and PANSS negative score were significantly and negatively associated with social media use.

**Conclusions:**

Less than one fourth of patients with schizophrenia and schizoaffective disorder use social media and may be suitable candidates for treatment programs supported by social media platforms , especially those of a young age and a low severity of negative symptoms .

**Disclosure:**

No significant relationships.

